# Integrative Characterization of Toxic Response of Zebra Fish* (Danio rerio)* to Deltamethrin Based on AChE Activity and Behavior Strength

**DOI:** 10.1155/2016/7309184

**Published:** 2016-11-23

**Authors:** Qing Ren, Tingting Zhang, Shangge Li, Zongming Ren, Meiyi Yang, Hongwei Pan, Shiguo Xu, Li Qi, Tae-Soo Chon

**Affiliations:** ^1^Institute of Environment and Ecology, Shandong Normal University, Ji'nan 250014, China; ^2^Management College, Ocean University of China, Qingdao 266100, China; ^3^Department of Biological Sciences, Pusan National University, Busan 609735, Republic of Korea; ^4^Ecology and Future Research Association, Busan 609802, Republic of Korea

## Abstract

In order to characterize the toxic response of zebra fish (*Danio rerio*) to Deltamethrin (DM), behavior strength (BS) and muscle AChE activity of zebra fish were investigated. The results showed that the average values of both BS and AChE activity showed a similarly decreased tendency as DM concentration increased, which confirmed the dose-effect relationship, and high and low levels of AChE and BS partly matched low and high levels of exposure concentrations in self-organizing map. These indicated that AChE and BS had slight different aspects of toxicity although overall trend was similar. Behavior activity suggested a possibility of reviving circadian rhythm in test organisms after exposure to the chemical in lower concentration (0.1 TU). This type of rhythm disappeared in higher concentrations (1.0 TU and 2.0 TU). Time series trend analysis of BS and AChE showed an evident time delayed effect of AChE, and a 2 h AChE inhibition delay with higher correlation coefficients (*r*) in different treatments was observed. It was confirmed that muscle AChE inhibition of zebra fish is a factor for swimming behavior change, though there was a 2 h delay, and other factors should be investigated to illustrate the detailed behavior response mechanism.

## 1. Introduction

Among contamination in substrate environment, water contamination with pesticides has significantly increased resulting from industrial and agricultural activities. Toxicity and residue problems are critical issues regarding water quality maintenance throughout the world [1, 2]. Pyrethroids are highly toxic to nontarget organisms such as honeybees, fish, and aquatic arthropods even at very low concentrations [3]. Deltamethrin (DM) is one of the most widely used pyrethroids and a common source of contamination in aquatic ecosystems [4]. It may impair biological communities, subsequently induce an unbalanced aquatic ecosystem, and then eventually cause unpredictable toxicity to humans and other biological organisms [5].

DM is a synthetic type II pyrethroid, which is more effective since it can not only affect the sodium channels of nerve filaments like other pyrethroids, but also affect the GABA receptors in the nerve filaments and affect chloride and calcium channels [6, 7]. DM could inhibit the synaptic membrane ATPase, delay Na^+^ channel closing time, and increase the number of opening Na^+^ channels [8]. Given that acetylcholine is one of the most widely distributed transmitter systems in the central and peripheral nervous systems of vertebrates, a general disruption of acetylcholine metabolism could impair various neuroendocrine or neurobehavioral processes related to the neurotransmitter substance. The toxicity was especially severe in fish [9].

A key enzyme that hydrolyzes the neurotransmitter acetylcholine in cholinergic synapses of both vertebrates and invertebrates, acetylcholinesterase (AChE), is strongly inhibited by pyrethroids at low concentrations [10]. For this reason, AChE has been widely used as a specific biomarker [11]. Consequently, this type of damage on neural systems would produce corresponding behavior changes in body movement. Many studies have found that a decrease in AChE activity may impair subsequent survival of the exposed organisms [12, 13]. It has been documented that inhibition of whole body AChE activity was a dominant factor for swimming behavior change to* Daphnia magna* under DDVP exposure [13].

The initial response of an individual fish to exposure is a possible change in its behavior, due to visual or chemoreception (olfaction, taste) damage [14]. Previous research has shown various response behaviors due to pyrethroids pesticides, such as loss of coordination of movement, jumping above the water surface, and moving in a circle [15, 16]. The Stepwise Behavior Response Model (SBRM) was proposed to address sequential behavior patterns during the course of response to the chemical [13, 17]. The model included no effect, stimulation, acclimation, adjustment (readjustment), and toxic effect, and similar behavior patterns were reported in macro-invertebrates [13, 17]. The SBRM postulates that an organism displays a time-dependent sequence of compensatory Stepwise Behavior Response in adapting to toxic effects on body motion during the course of exposure to pollutants [18].

The relationship between the continuous inhibition of AChE activity and behavior of fish in contaminants has not been studied extensively. The zebra fish* (Danio rerio)* has become an important vertebrate model organism in OECD guidelines, such as developmental and genetic research and pharmacological studies [19]. It is recommended as a standard model aquatic organism for toxicity tests [20] and is very sensitive to the external changes caused by chemical composition in the environment. It has been frequently used as a representative of freshwater fish in toxicological assessment of the toxin [21]. In this study, we (1) investigate the toxic effects of the DM on concurrent behavior responses and inhibition of muscle AChE of zebra fish* (Danio rerio)*, (2) comparatively characterize the two responses as the time progressed, (3) integrate behavioral and physiological toxic effects in association with behavior strength of zebra fish, and (4) analyze/compare both physiological and behavioral parameters.

## 2. Materials and Methods

### 2.1. Species and Chemicals

The zebra fish* (Danio rerio)* has been widely used for toxicological testing [22]. The populations for the experiment were obtained from the China Zebra Fish Resource Center in Wuhan, China. The populations were cultured over three generations under constant filtration with nonchlorinated water (hardness based on CaCO_3_ 250 ± 25 mg/L, pH 7.8) in our laboratory. The stock population was maintained at 26 ± 2°C with a 16 h light period (4000 lx) and a 8 h dark period (lights on at 5:00 am). The populations were fed with a commercial flake fish food (Trea®, Germany) twice daily at 8:30 am and 4:30 pm. In all experiments, males and females about 3 cm long were selected randomly to exclude gender impact [23]. Females in ovarian cycle were removed before selection. Feeding was stopped 24 h before treatment.

DM (technical grade, 95% purity) was purchased from the China National Standard Sample Center. The solvent, dimethyl sulfoxide (DMSO), was purchased from Biosharp Corporation. The exposure concentration of DMSO within the water was lower than 0.5%, which would neither cause acute toxicity nor affect the motility of zebra fish [24]. Acetylthiocholine iodide (ATCh), 5, 5-dithio-2, 2-nitrobenzoic acid (DTNB), and bovine serum albumin (BSA) were purchased from Sigma (Sigma-Aldrich Corporation, St. Louis, MO, USA) for measuring toxicity. All of the chemicals were of analytical grade (95% purity).

### 2.2. Determining AChE Activity

**Figure 1:**
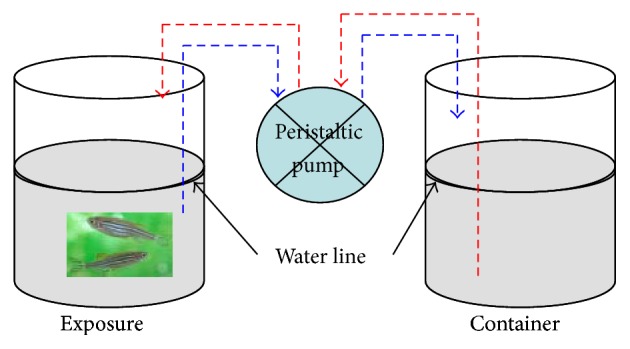
Dynamic exposure system for zebra fish. Arrows of dotted lines show the direction of water flow through the multichannel peristaltic pump: red-inflow and blue-outflow.

48 h LC_50_ of DM to zebra fish was 5.20 *μ*g/L with a 95% confidence interval (3.99–7.04 *μ*g/L, Table 1) in a dynamic exposure system using 5000 mL beakers under the same condition as for culture (Figure 1). 5.20 *μ*g/L was taken as one toxic unit (1.0 TU). Muscle AChE activity was measured after 48 h of continuous exposure to 0 TU, 0.1 TU, 1 TU, and 2 TU DM in the same exposure system using 5000 mL beakers (Figure 1) and a flow rate of about 2 L/h. Nonchlorinated water (hardness based on CaCO_3_ 250 ± 25 mg/L, pH 7.8, and temperature 26 ± 2°C) was used. In this experiment, there were three replicate exposure beakers for each group, and 100 individuals were exposed in each exposure beaker. No food was provided to test organisms during the experiment. Three individuals in each treatment were taken for observation at exposure times of 0 h, 0.5 h, 1 h, 2 h, 4 h, 8 h, 16 h, 32 h, and 48 h. The whole muscle from the caudal peduncle was dissected out and was used to prepare homogenate fraction. The samples were stored at −80°C.

81 mL 0.1 M disodium hydrogen phosphate and 19 mL 0.1 M sodium dihydrogen phosphate were mixed and then diluted with deionized water to 100 mL to prepare phosphate buffer (0.1 M, pH 7.4). Homogenates were prepared in ice-cold phosphate buffer using a mechanically driven Teflon fitted Potter-Elvehjem homogenizer for 2 min at 3000 r.p.m in ice until total disruption of muscle. The homogenates were then centrifuged at 12,000 r.p.m for 20 min at 4°C [25]. The supernatant was used as an enzyme source for measuring the activity of AChE. AChE activity in the homogenates was detected as follows: 50 *µ*L enzyme and 50 *µ*L ATCh (5 mM final concentration) were incubated at 30°C for 15 min in a final volume of 0.1 mL. The reaction was stopped with 0.125 mM DTNB-phosphate-ethanol reagent in 0.9 mL (12.4 mg of DTNB dissolved in 125 mL 95% ethanol, 75 mL distilled water, and 50 mL 0.1 M phosphate buffer, pH 7.5) as the thiol indicator. The color was detected immediately at 412 nm using an ELISA (Infinite M200) [26]. Based on the Bradford Protein Assay of the protein concentration of enzymatic extracts [27], the AChE activity was expressed as nmol/min/mg protein. The muscle AChE activity (% of control) was used to analyze the effects of DM on the AChE activity.

### 2.3. Measuring Behavior Strength (BS)

Four concentrations (0.0 TU, 0.1 TU, 1 TU, and 2 TU DM) were used to evaluate behavior responses of zebra fish. The motility of test organisms was observed and defined as behavior strength (BS) (for definition of BS, see [28]), which was continuously recorded for 48 h with the flow-through test chambers (7 cm long, 5 cm in diameter) in an online monitoring system (OMS) built in the Research Center for Eco-Environmental Science, Chinese Academy of Sciences. The test chamber was closed off with nylon nets (250 *μ*m) on both sides [28].

Three individuals were placed within each chamber and the flow rate was controlled at about 2 L/h, which has no effect on the motility of test organisms [13, 29]. No food was provided during the observation period. Nonchlorinated water (hardness based on CaCO_3_ 250 ± 25 mg/L, pH 7.8) was used. Three replicates were used to measure behavior strength in each group (concentration). BS was sampled automatically every second, and the average behavior strength per 6 min was used to analyze behavior response. The values were normalized between 0 (no motility) and 1 (full activity) to illustrate the behavioral response differences of zebra fish according to sampling times and TUs [18].

### 2.4. Data Analysis

Though the systems for AChE activity detection and behavior strength monitoring were different, the effects of different exposure systems on the physiological/ecological changes of zebra fish could be ignored due to the following methods: 1st, test individuals of similar body size were selected randomly; 2nd, the two dynamic exposure methods were running under the same conditions; 3rd, the flow rate controlled by the multichannel peristaltic pump was the same.

Behavior strength (BS) and AChE activity results were tested using one-way analysis of variance (ANOVA) and multiple comparisons [30]. The self-organizing map (SOM) was used to classify movement patterns by training the continuous movement data of BS [31]. The SOM was trained to show patterns of BS and AChE activity in association with experimental conditions using the SOM Toolbox developed by the Laboratory of Information and Computer Science, Helsinki University of Technology in MATLAB environments [32].

Integration of time series BS values was also used in this study to reveal toxic responses of test organisms as the time progressed [31]. After integration, the data were statistically fitted to linear model regression. Subsequently residual curves of integral BS values from the linear fitting were produced to define behavior activity during the course of behavior responses. In order to illustrate time-delayed toxic effect, correlation coefficients (coefficient* r* and significance* p*) were obtained according to time difference in sampling times (log scale). MATLAB© 2009 (1984–2009 The MathWorks, Inc.) was used to analyze the time-difference correlation of AChE activity and BS. The linear regression analysis was used to get the time delay correlation equations of both muscle AChE activity (% of control) and zebra fish BS.

## 3. Results and Discussion

### 3.1. Overall Toxicity Patterns

**Figure 2:**
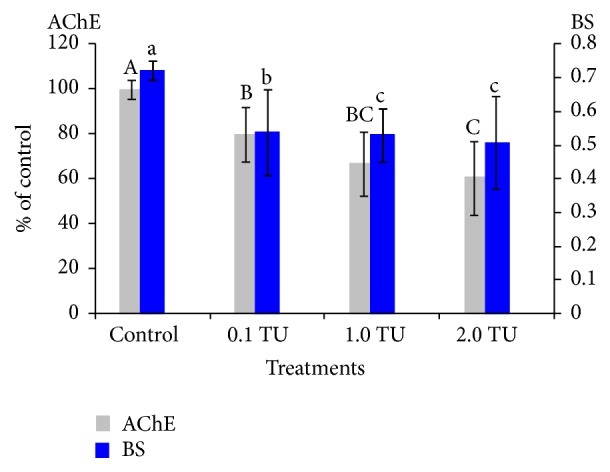
Comparison of BS and muscle AChE activity during 48 h exposure to different treatments. The mean values during 48 h exposure are shown as mean ± S.D. *n* = 3. The gray columns represent the mean values for AChE and the blue columns BS; the solid lines indicate Standard Deviation. Different letters represent statistical significance (*p* < 0.05) according to multiple comparisons. Capital letters showed significant differences in AChE inhibition among different treatments and lower case letters significant differences in BS. AChE activity in control at the beginning of the experiments is regarded as 100%.

Changes in both zebra fish BS and muscle AChE activity with different DM treatments with control are shown in Figure 2. The mean values for both BS and AChE activity during 48 h exposure showed a similar tendency to decrease as DM concentration increased (control > 0.1 TU > 1 TU > 2 TU), which confirmed the dose-effect relationship. However, slight difference could be observed in the two measurements according to concentration: although statistically significant differences were observed with TUs, the toxic effect in control and 0.1 TU groups was not substantially different for AChE activity compared with BS (Figure 2). Overall differences appeared to be relatively smaller in AChE activity.

**Figure 3:**
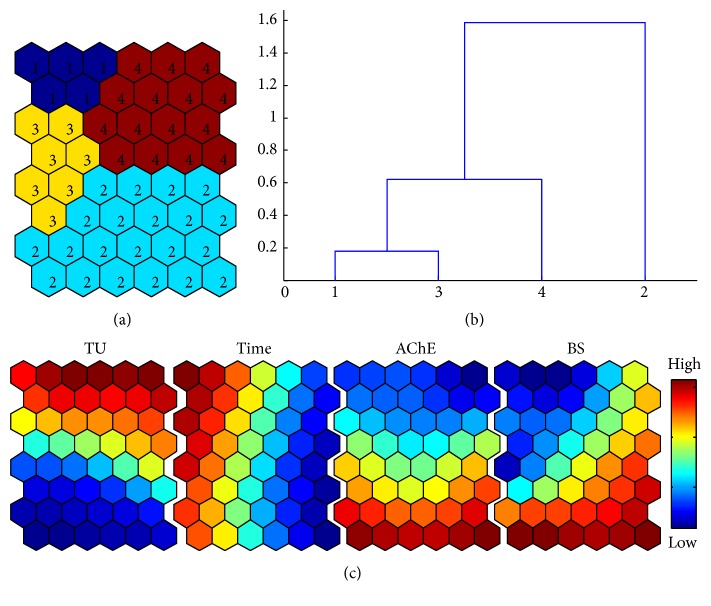
SOM patterning on toxicity variables (BS and AChE activity) and experimental conditions (TUs and observation times). (a) Four clusters according to the SOM training; (b) cluster distances based on dendrogram by Ward's linkage method; and (c) profiles of variables on component of map of SOM.

Considering experimental conditions (TUs and observation times (Time)) as variables, associations with the toxicity effects on BS and AChE were presented by the self-organizing map (SOM) (Figure 3). According to Ward's linkage method, four clusters were identified (Figures 3(a) and 3(b)). Along the vertical direction, concentrations showed a clear gradient with higher levels in the top area of the map (clusters 1 and 4). AChE also showed a vertical gradient but negatively associated with the TUs, indicating a negative relationship with TUs. BS also showed a similar trend, but a slight difference was observed compared with AChE. A diagonal gradient was observed with minimum and maximum levels at the top left and bottom right corner, respectively (bottom right panel, Figure 2(c)). High and low levels partly matched low and high levels of TU. This indicated that AChE and BS had slight different aspects of toxicity although the overall trend was similar.

It was also noteworthy that BS showed a relatively high level at the top right corner that matched AChE with the minimum level. This area was also in accordance with maximum TU and minimum level of Time; there were cases where the BS values at relatively high levels corresponded with maximum values of TU in the early phase. This suggested that BS was not much influenced by the chemical initially. The experimental condition of time separately showed a horizontal gradient indicating that Time is not much associated with TU and AChE. BS, however, was partly associated with Time, especially in the top left area.

### 3.2. Time Changes in Toxicity

**Figure 4:**
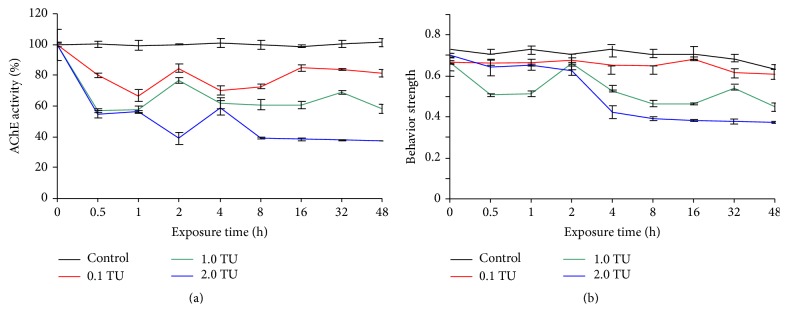
The effects of DM on muscle AChE activity and BS of zebra fish as the time (log scale) progressed during 48 h exposure. The curves in different colors showed the mean values of muscle AChE activity and BS, and the vertical black lines showed the Standard Deviation for muscle AChE activity and BS. (a) AChE activity (% of control) inhibition and (b) behavior strength.

Although the overall toxicity trend was similarly expressed in average values as shown in Figure 2, time changes in BS and AChE activity were notably variable (Figure 4). The levels of both AChE activity and BS were stable in the high level during the exposure period in the control. For AChE activity, a rapid decrease was observed in the initial period between 0 h and 0.5 h across different treatments (Figure 4(a)) whereas BS values remained remarkably high in the early period (Figure 4(b)), suggesting that BS was little influenced by the chemical in the initial exposure phase (Figure 3(c)). However, immediate responses were observed in AChE. After 1 h, the toxicity levels of AChE were briefly stable across TUs. At 2 h, however, a sudden increase (with 0.1 TU and 1.0 TU) and a decrease (with 2.0 TU) were observed in AChE activity. This may be related to photoperiod, which was attributed to the circadian rhythm caused by some acetyltransferases [33], but the real reason is currently obscure.

In comparison with AChE, however, the BS maintained somewhat high levels in the initial period for all treatments and then showed a gradual decrease in 1.0 TU and 2.0 TU treatments (Figure 4(b)), while it was stable in all time periods in 0.1 TU. This confirmed the area of the component SOM showing relatively high levels of BS values in the early phase (Figure 3). It was noteworthy that BS values were reversed in the early period between 0 h and 1 h with the highest 2.0 TU while 1.0 TU showed the minimum BS in this period.

Later, a gradient in activity was observed, showing lower BS responding to higher TUs accordingly (Figure 4(b)). Overall, more fluctuation was observed showing peaks in BS values compared with AChE activity (Figure 4(b)). BS showed peaks at 2 h with 1.0 TU. BS values decreased substantially in 1.0 TU and 2.0 TU from 4 h (Figure 3(b)). It was noteworthy that the trend of BS and AChE became similar later. We hypothesized that the toxic effects of the chemical first affect the physiology (a significant decrease of 20%) and affected the external behavior later (behavior strength began to decrease after two hours (20%)), and this may cause a time delay in the effect on AChE.

**Figure 5:**
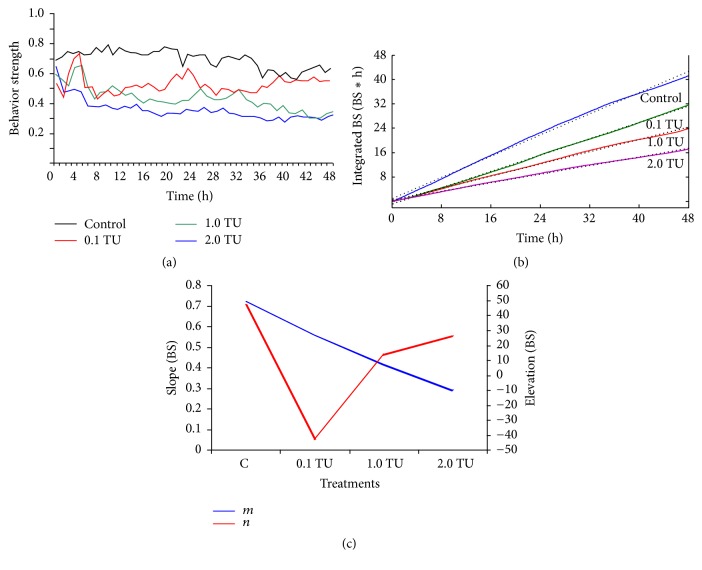
Integrated BS values and behavior activity (BA) after linear fitting when test organisms are exposed to DM. (a) The time series BS (measured every 6 min during 48 h exposure); (b) integrated BS values as the time progressed in different TUs. The black lines indicate linear regression fitted to the integrated BS values, and the colored lines indicate four concentration linear fitting lines, respectively; and (c) slopes, “*m*”, and elevations, “*n*”, for linear regression fitting to integral BS values. There are no significant differences (*p* < 0.05) of both “*m*” and “*n*” across different concentrations according to multiple comparisons.

According to integration of the time series BS values [31], integrated BS values were obtained as the time progressed across concentrations (Figure 5). The highly fluctuating nature of the original BS values was filtered efficiently to show linear development of toxic behavior responses (Figure 5(a)). The highest slope was observed for the control (i.e., least toxic effect) (Figure 5(b)), while the slope decreased as concentration increased. Slopes (*m*) and elevations (*n*) in the fitted linear regression models were presented in Figure 5(c). Due to decrease in BS values, slopes (*m*) of lines decreased as the concentration increased and showed no significant difference (*p* < 0.05) between concentrations. In the case of elevations (*n*), a gradient along the increase in concentrations was not clearly observed (*p* > 0.05).

**Figure 6:**
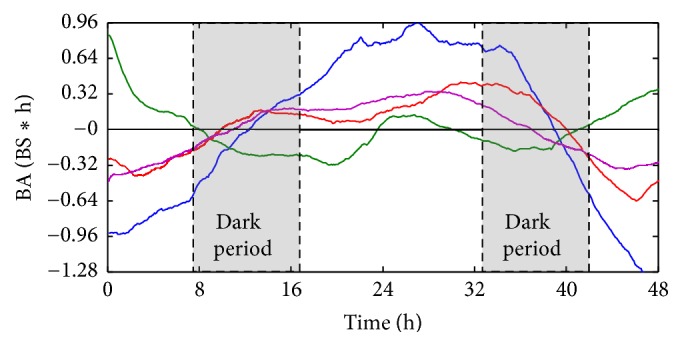
Behavior activity (BA) values of zebra fish in different treatments across the 48 h exposure. The blue curve shows the control behavior responses of zebra fish, the green curve in 0.1 TU DM, the violet curve in 1.0 TU DM, and the red curve in 2.0 TU DM. The gray area shows the dark period and the unshaded area the light period.

Behavior activity was defined as the difference between integrated BS and linear fitting [31] (Figure 6). If behavior activity is in the positive range, the test organisms had greater BS compared with time-averaged BS values obtained from the linear fitting. Test organisms would be in the “active” state. If behavior activity is in the negative range, test organisms would be in a less active state. It was noteworthy that the crossing times of behavior activity on the *x*-axis between positive and negative values of behavior activity on the *y*-axis were commonly observed across concentrations (Figure 6). The crossing times from negative to positive (or vice versa) values in Figure 6 were indicative of the response state of test organisms to chemicals. If the behavior activity value is positive, the organisms are in the active state, indicating that they adjust themselves to increase activity against intoxication effects. If the behavior activity value is negative the reverse situation would occur, indicating lower activity due to acclimation of the toxic effect. These two phases could be considered as acclimation and adjustment, respectively, as defined for BS modes [18]. Inclining and declining phases were clearly observed between peaks. During the period of declining phase, test organisms would have a tendency in losing behavior activity values continuously until the values reach the minimum peak, and this declination period would represent intoxication due to a continuous decrease in behavior activity values in test organisms. A continuous increase in the behavior activity values would indicate recovery from intoxication. New states of test organisms were defined as intoxicating and recovering tendencies corresponding to the inclining and declining phases of behavior activity values, respectively, based on the experimental conditions in this study [31].

Study on zebra fish showed that circadian rhythms can be initiated and maintained in the absence of the suprachiasmatic nucleus (SCN) and other tissues in the ventral brain, though the SCN may play a decisive role in the regulation of the amplitude of rhythms in the absence of environmental cues [34]. The findings on clock genes and their regulation are well documented in the brain and pineal independently in several vertebrates including zebra fish [35, 36].

Melatonin, first detected from the bovine pineal gland [37], is known to be present in many organs, tissues, and organelles [38]. Clock genes regulate the biosynthesis of melatonin and therefore indoleamine is the potential candidate for mediating circadian process in animals [39]. Melatonin is produced only in darkness irrespective of the diurnal or nocturnal habit of the organism [39]. Melatonin biosynthesis is conserved [40] and involves four enzymatic steps using tryptophan as the precursor. These four enzymes are also involved in the production of melatonin in the teleost [35].

In the control group, clear variation was observed across the 0 line on the *y*-axis. It was noteworthy that the behavior activity curve showed a gradual increase in the light period between around 10 h and 36 h (Figure 6). Although the positive behavior activity values were shown in the photophase, behavior activity did not increase in the light period in the early and late period of observation (Figure 6). This indicated that the increase in behavior activity values was not due to circadian rhythm in the control group.

After exposure to the chemical, however, behavior activity patterns were substantially different. With the minimum concentration of 0.1 TU, it was remarkable that the positive side was additionally observed in both the early and late phase. It was also noteworthy that the behavior activity curve increased to the positive side in the middle of the light phase although the period was relatively short. In addition the curve decreased consistently in the dark period at around 8 h–16 h and 32 h and 40 h at 0.1 TU during the observation period. Considering the ups and downs in BS curve matched to photo- and scoot-phase, this may suggest a possibility of reviving the circadian rhythm in test organisms after exposure to the chemical.

However, as the TU increased, this type of rhythm disappeared. With an increase to 1.0 TU and 2.0 TU substantial differences in behavior activity patterns were observed. Overall changes in behavior activity were similar to the control (Figure 6). Instead of stimulation at 0.1 TU, acclimation was observed initially by showing negative behavior activity values (Figure 6). Behavior activity values increased afterwards and remained at a high level until the end of the next dark period. This indicated the rhythm disappeared again at higher concentrations but the mechanism is currently unknown.

### 3.3. Time-Difference Correlations between AChE and BS

To interpret the correlation results, we first checked the correlation coefficient *r* to see how much they correlated (*r* < 0.3, poor correlation, 0.3 < *r* < 0.5 moderate, and *r* > 0.5 high correlation). Then we checked* p* value to see whether these two variables are correlated significantly (*p* < 0.05). When* r* is high (absolute *r* > 0.5) with significance (*p* < 0.05), it indicates the data correlation is significant [41]. With different time delay (−1 to 5 sampling-time delays (log scale)) shown, 3 log scales had greatest relationship among them with high correlation coefficients (*r*) 0.71, 0.73, and 0.94 for 0.1 TU, 1.0 TU, and 2.0 TU, respectively (Table 2), and the average values (0.79) were much higher than other log scale changes, for example, 0.44 in −1 log scales, 0.54 in 0 log scale, 0.10 in 1 log scale, 0.17 in 2 log scales, 0.54 in 4 log scales, and 0.56 in 5 log scales. Meanwhile, the correlation significance (*p*) in 3 log scales ((BS + 0)−(*t* + 3)) was higher than others, especially for the 0.1 TU treatment. According to visual observation, high levels of BS were maintained until 2 h, whereas AChE activity immediately decreased after exposure to the chemical (Figure 4). After 2 h, a decrease in BS was observed across TUs. This suggested a possibility of delayed effect on behavior after the toxic effect initially occurred on AChE activity. For these two reasons, we chose minus the front 3 log scales of all BS TUs and the last 3 log scales of all relative AChE activity concentrations to analyze the relationship between AChE and BS.

**Figure 7:**
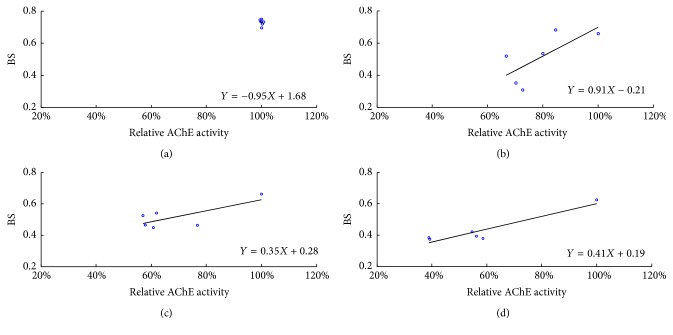
Time-difference correlations between BS and AChE activity. All the BS measurements were 3 sampling-time delays (log scale) in relation to all the relative AChE activity minus last log scales. Scatter grams and line of regression between BS and AChE activity for (a) control; (b) 0.1 TU; (c) 1.0 TU; and (d) 2.0 TU.

In the control group, scatter points all concentrated. In the experimental groups, all three concentrations were roughly in a straight line. The correlation analysis suggests that the changes of BS with each treatment show a positive relationship with AChE activity inhibition when the BS concentrations were minus the front 3 log scales and minus the last 3 log scales of AChE (Figure 7). For 2.0 TU treatment, the correlation coefficient (*r*) is 0.94. For other treatments (0.1 TU and 1.0 TU), the correlation coefficients are 0.71 and 0.73, respectively, which could support the good correlation of both parameters. For 2.0 TU, there was significant difference (*p* < 0.01). The results of both behavior responses and AChE activity for the four treatments after minus 3 log scales showed the tendency was similar to each other. All could be described by the Stepwise Behavior Response Model including no effect, stimulation, acclimation, adjustment (readjustment), and toxic effect [13, 17], which meant the loss of nerve conduction ability was correlated directly with the toxic effects on behavior as reported by Xuereb et al. in 2009 [42]. Bayne [43] had proposed that the adaptive response of fish to stress can be divided into 3 stages. The first stage refers to the changes in neuroendocrine activities; the second stage includes a series of physiological, biochemical, and immunological reactions induced by the first stage of neuroendocrine activities; and the third stage would be subsequent changes in other systems, including changes in behavior and development disease resistance mechanisms. The current result may indirectly support the third step: there was a time delay in the production of response behaviors after neuroendocrine effects.

It proved our hypothesis that there is 3 log scales' delay when AChE inhibition causes a change in swimming behavior during DM exposure. This may also be the reason that the BS values remained high in the early period (Figure 4(b)) and decreased significantly (20%) 2 h later.

## 4. Conclusion

In the present study, we analyzed the toxic response of zebra fish to DM based on behavior strength and muscle AChE activity. The results showed that the circadian rhythm occurred with 0.1 TU treatment, and this rhythm disappeared at higher concentrations (1.0 TU and 2.0 TU). Previous research showed that AChE inhibition was an important factor for swimming behavior changes of* D. magna* under DDVP exposure [13]. Our results also confirmed that muscle AChE inhibition in zebra fish is a factor disorder in swimming behavior and there is also a 2 h delay between AChE inhibition and the behavior disorder. In the future, other organs (brain, gill, and liver) should be investigated to determine whether there are time delay effects under the same conditions.

## Figures and Tables

**Table 1:** 48 h LC_50_ of DM to zebra fish *(Danio rerio)*.

Chemicals	LC_50_-48 h	95% confidence interval	Regression equation	*R*
DM	5.20 *μ*g/L	3.9–7.04 *μ*g/L	*y* = 1.25*x* − 1.50	0.94

**Table 2:** Time-difference correlation (correlation coefficient (*r*) and significance (*p*)) with different time delay of BS at each concentration.

Data delay methods	Correlation coefficient (*r*) and significance (*p*)	C	0.1 TU	1 TU	2 TU
(BS + 1)−(*t* + 0)	*r*	−0.22	−0.18	−0.36	−0.77
	*p*	0.60	0.68	0.38	0.03
(BS + 0)−(*t* + 0)	**r**	−**0.16**	**0.10**	**0.87**	**0.66**
	**p**	**0.68**	**0.79**	**0.00**	**0.01**
(BS + 0)−(*t* + 1)	*r*	0.26	−0.32	−0.17	0.69
	*p*	0.53	0.44	0.69	0.58
(BS + 0)−(*t* + 2)	*r*	0.05	−0.01	−0.24	0.76
	*p*	0.91	0.99	0.60	0.05
(BS + 0)−(*t* + 3)	**r**	−**0.22**	**0.71**	**0.73**	**0.94**
	**p**	**0.67**	**0.11**	**0.10**	**0.01**
(BS + 0)−(*t* + 4)	*r*	−0.26	−0.06	0.79	0.88
	*p*	0.67	0.92	0.11	0.05
(BS + 0)−(*t* + 5)	*r*	0.80	0.26	0.46	0.94
	*p*	0.20	0.74	0.54	0.06

“(BS + 1)−(*t* + 0)” means the BS minus the last 1 log scale in each concentration and the normal AChE minus the last 1 log scale in each concentration; “(BS + 0)−(*t* + 1)” means the normal BS in each concentration and the AChE minus the last 1 log scale in each concentration.
